# A first case of ileocecal resection using a Senhance Surgical System in Japan

**DOI:** 10.1186/s40792-020-00859-0

**Published:** 2020-05-07

**Authors:** Hiroka Kondo, Shigeki Yamaguchi, Yasumitsu Hirano, Toshimasa Ishii, Nao Obara, Liming Wang, Masahiro Asari, Takuya Kato, Tetsuyoshi Takayama, Hirofumi Sugita, Shinichi Sakuramoto, Isamu Koyama

**Affiliations:** Department of Gastroenterological Surgery, Saitama Medical University International Medical Center, 1397-1 Yamane, Hidaka-shi, Saitama, 350-1298 Japan

**Keywords:** Colon cancer, Robot-assisted surgery, Senhance

## Abstract

**Background:**

Several manufacturers are in the process of developing various innovative systems and expect more options in the robot market. One of the latest systems, the Senhance® platform (TransEnterix Surgical Inc, Morrisville, NC, USA), has already been introduced in Europe and has also been approved for clinical use in Japan. We report the first case of colorectal resection using Senhance in Japan.

**Case presentation:**

The patient was a 79-year-old Japanese man who visited a previous physician for positive fecal occult blood. Upon close inspection, the preoperative diagnosis was cT2N0M0 stage I. We performed surgery using Senhance. The operation time was 198 min, and the estimated amount of bleeding was 10 g. He was discharged after surgery without any major complications. However, it is also true that the operability of the conventional port arrangement was poor during the surgical operation.

**Conclusion:**

We report the first Senhance-assisted ileocecal resection for colorectal cancer in Japan. In the future, we would like to find more ways to use it by accumulating more cases.

## Background

Until recently, robot-assisted surgery had referred to the da Vinci Surgical System [1]. However, several manufacturers are in the process of developing various innovative systems and expect more options in the robot market [2]. One of the latest systems, the Senhance platform (TransEnterix Surgical Inc, Morrisville, NC, USA), has already been introduced in Europe and has also been approved for clinical use in Japan. The Senhance has advanced vision features and control with tactile feedback. The reusable device offers the same low operating costs as traditional laparoscopic surgery, which is very important in the Japanese health care system. Reports of Senhance-assisted surgery have begun to appear [3, 4], but there are few reports on its clinical use for colorectal cancer internationally [5–9]; therefore, we report on our experience in this hospital.

## Case report

**Fig. 1:**
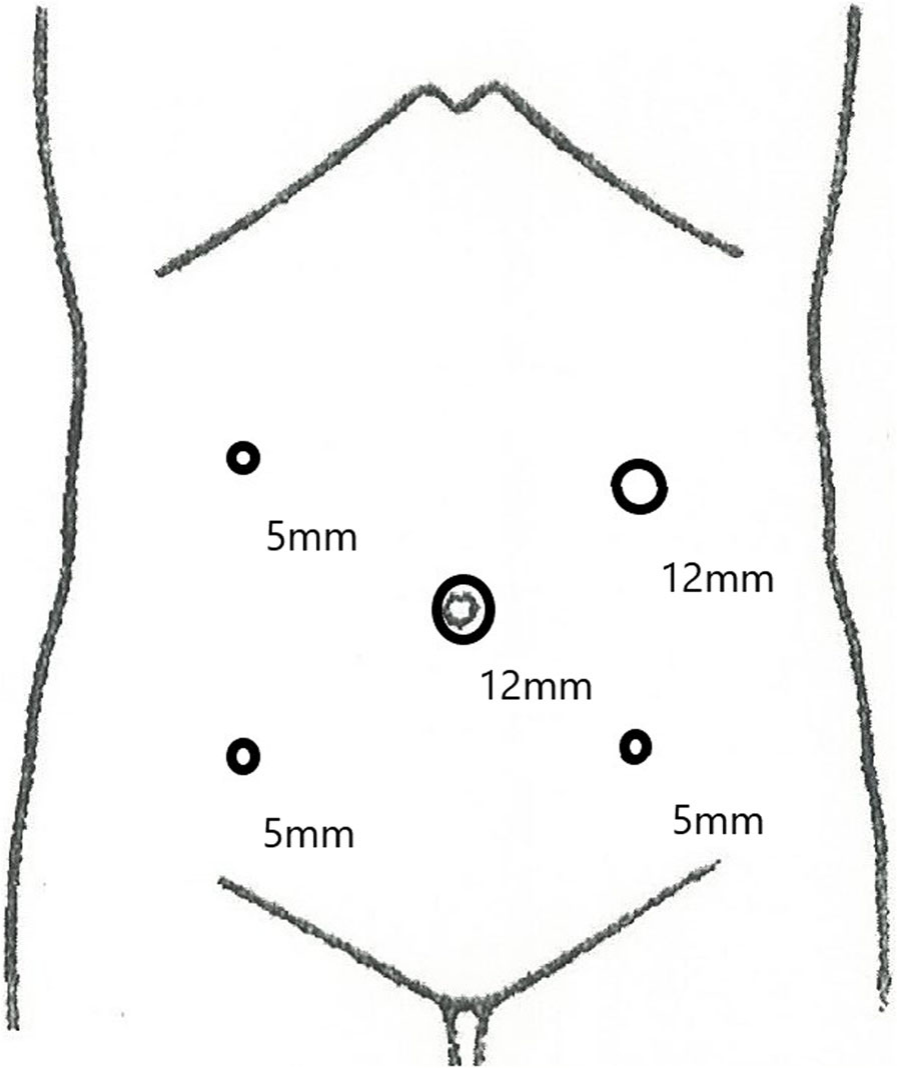
Port placement during conventional laparoscopic ileocecal resection

**Fig. 2:**
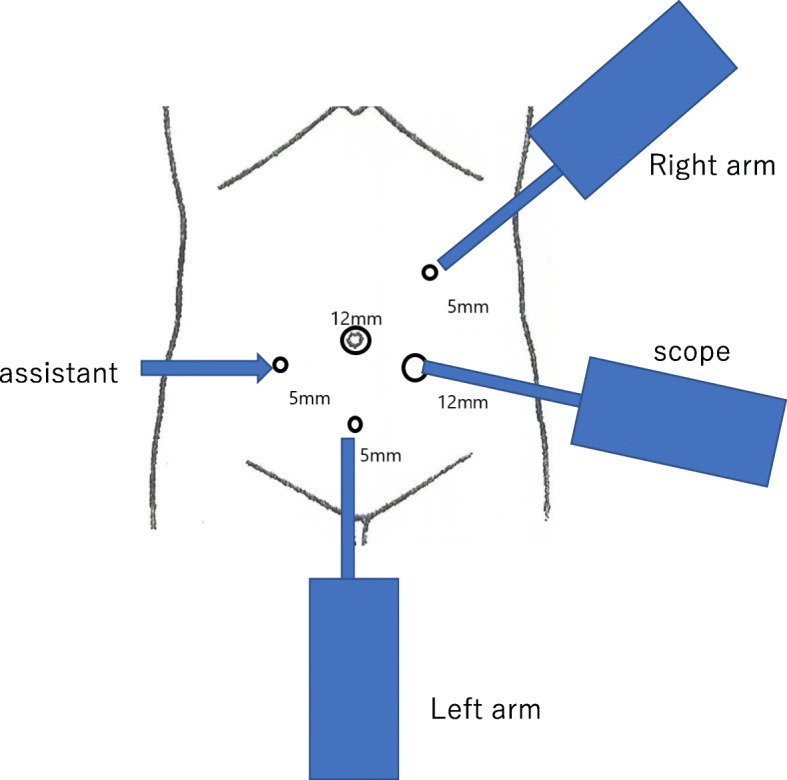
Port placement at the time of Senhance-assisted ileocecal resection

**Fig. 3:**
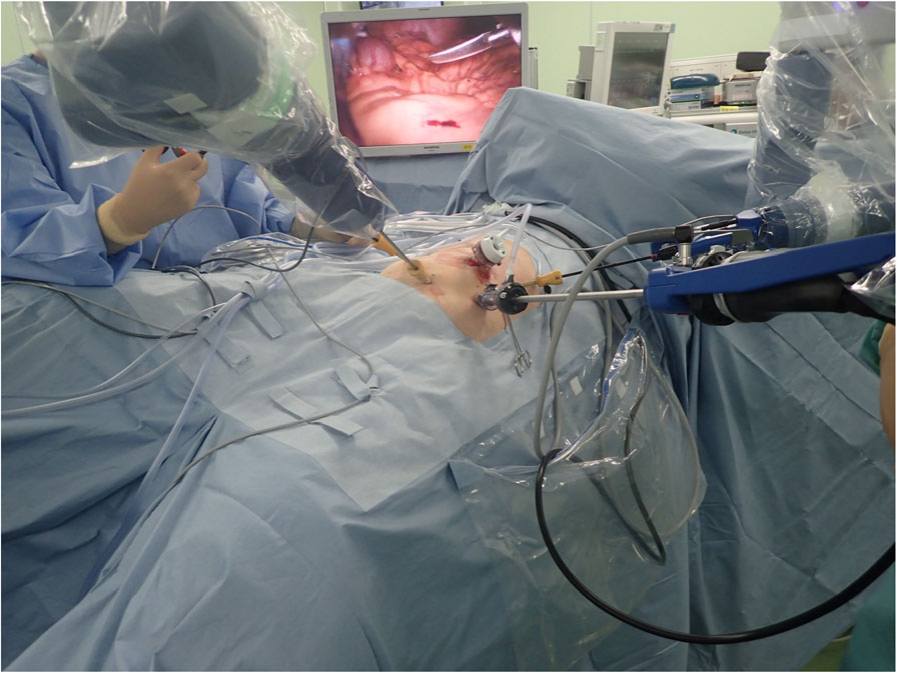
Operative room installation—patient positioning on table: ileocecal resection

The patient was a 79-year-old Japanese man who visited a previous physician for positive fecal occult blood. Colonoscopy revealed a type 2 mass in the cecum, and a biopsy revealed Tub1, so he was referred to our hospital. He was 149 cm tall and weighed 51 kg, with a body mass index (BMI) of 23.2 kg/m^2^. His performance status (PS) was 0, his history included chronic obstructive pulmonary disease (COPD), and his American Society of Anesthesiologists (ASA) PS was 3. Computed tomography revealed no obvious distant metastasis, and the preoperative diagnosis was cT2N0M0 stage I. We performed surgery using Senhance. In our hospital, it is conventionally implemented with 5 ports as shown in Fig. 1. This time, the port arrangement was changed as shown in Fig. 2. With reference to the port arrangement for left colon surgery in the report of Spinelli et al. [9], this arrangement was determined by arranging for the right colon (Fig. 3). The operation time was 198 min, and the estimated amount of bleeding was 10 g. The docking time and the console time were not measured but required approximately 30 to 40 min to dock. The patient’s body was placed in the lithotomy position, with the head and the left side of the patient slightly lower. First, the pedicle of the ileocolic artery and vein was grasped and towed, the mesentery was incised caudally, and the mesenteric mobilization was started. After dissection around the root of the ileocolic artery and vein, the blood vessel was treated with scissors after double clipping. The exercise was completed toward the hepatic flexure. Subsequently, the small intestine was moved cranially, and a peritoneal incision was made outside the ascending colon. Finally, the hepatic flexure was activated as the head was elevated, and the console procedure was completed. A small incision was made in the umbilical 12-mm port insertion, the ileocecal part was pulled out and excised outside the body, and a functional end-to-end anastomosis was performed to return the anastomosis into the body. Even in the conventional laparoscopic ileocecal resection, we perform resection anastomosis outside the body from the small umbilicus in our department. The ileocecal region can be easily pulled out from a small laparotomy wound. If intraperitoneal anastomosis is performed, the operator’s right port must be 5 to 12 mm for inserting the stapler. Therefore, it was judged that there is little merit to perform an intraperitoneal anastomosis. Although bloody stool was temporarily observed after the operation, the condition improved only with conservative treatment. The diet was resumed on the sixth postoperative day, and the patient was discharged on the ninth postoperative day.

## Discussion

**Fig. 4:**
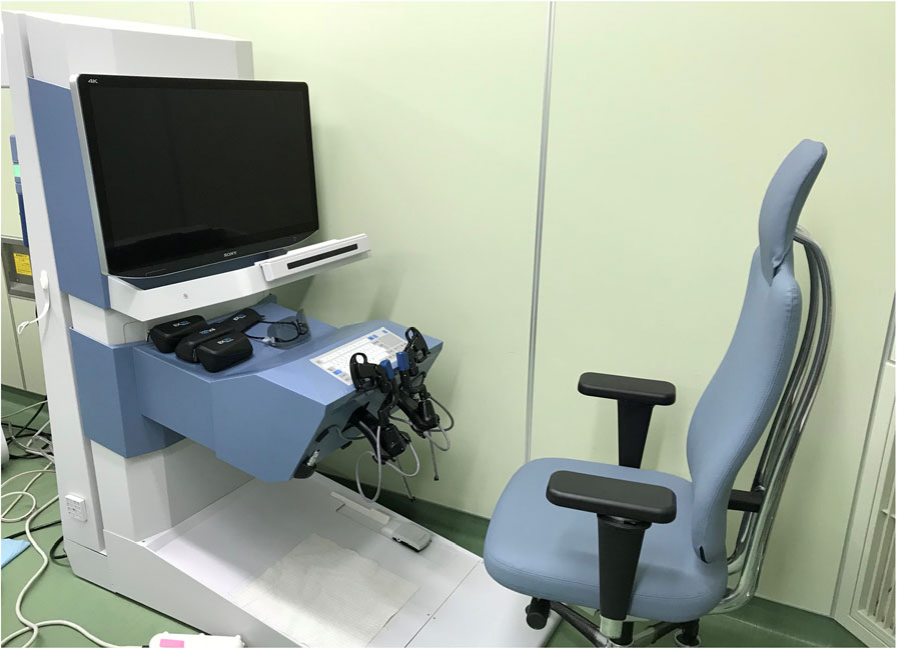
A system of the remote control unit called a cockpit

**Fig. 5:**
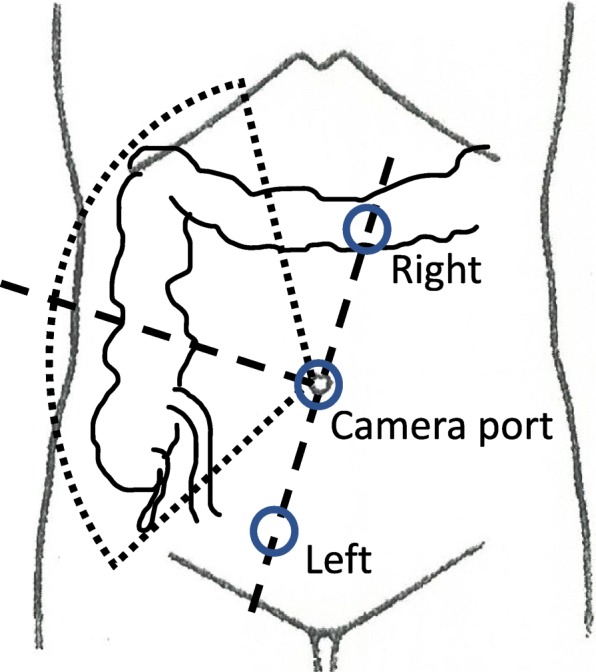
Ideal port placement we think

**Fig. 6:**
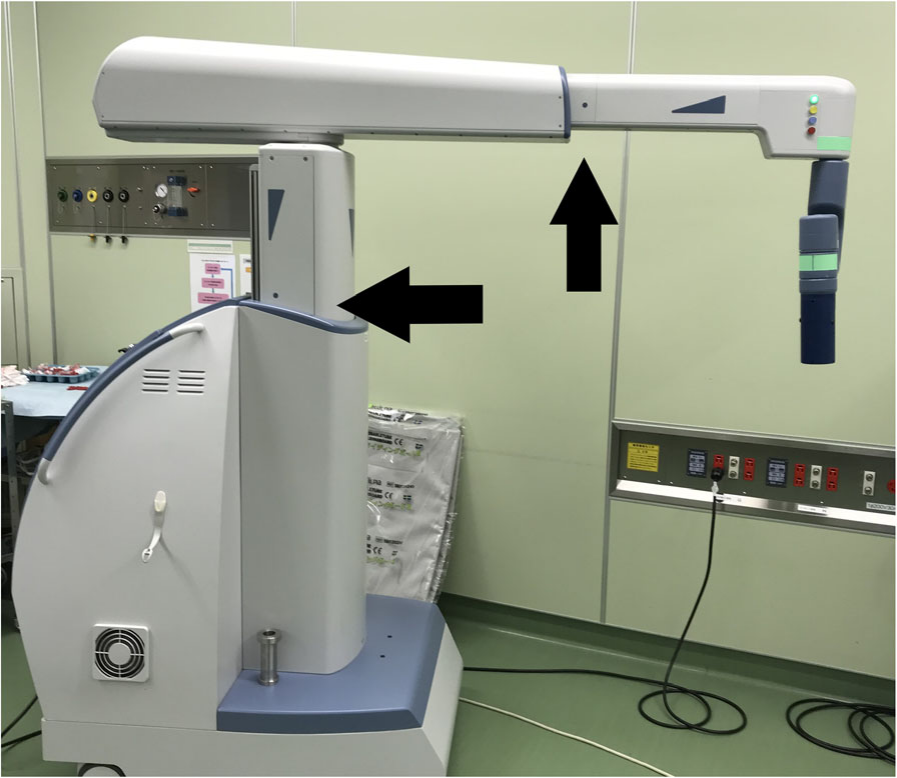
It is important to set the position of each arm so that it is at the middle point (→) when docking for the first time

The Senhance was developed by the Italian company Sofar and was initially named ALF-X. After negotiations with the Transenterix Company based in the USA, the decision was made to change the product’s name to the Senhance Surgical System. The Senhance system has a remote control unit called a cockpit (Fig. 4), a robotic arm which provides three-dimensional (3D) high-definition (HD) monitoring, an infrared-ray eye-tracking system, a foot pedal, a keyboard, a touch pad, a connection node, and a reusable laparoscope apparatus. This system has several unique functions. It is possible to make these functions fit with use of the optional 3D optical system. Surgeons have the ability to judge the location of objects in the surgical field by using an eye-tracking camera. In addition, the operating handle of the apparatus has tactile feedback, which will be of help during suturing and cutting. It has been reported that 3D vision was significantly faster than the two-dimensional (2D) vision available during da Vinci-assisted surgery [10], and 3D vision may help to perform the surgical operation more easily. The surgeon takes a seat where an adjustment is possible for perfect positioning and chooses an erect posture. People present in the operating room can see the same monitor as the surgeon [2]. This Senhance-assisted surgery was our first experience. We practiced in the dry lab and then the operating room, but the characteristics of this type of surgery were different from conventional laparoscopic surgery. For the Senhance-assisted surgery, we thought it would be better to change the trocar placement. For example, as shown in Fig. 5, it is ideal to arrange the camera port and the operator’s left and right ports on a 90° line with respect to the center of the range to be operated, and add an assistant port if necessary. In addition, if docking is not performed in consideration of the extension restriction of each arm, intraoperative movement restriction occurs, so it was necessary to confirm that each arm was near the center point at the time of docking (Fig. 6). If this step is neglected, the surgical operation on the console will soon reach its limit. Conversely, if each arm can be docked to be at the middle point of the operation range during the first setting, the operation on the console becomes easy, and the operator can perform the operation in a comfortable posture. This Senhance-assisted ileocecal resection is the first case in our department, and it took time to set up. However, the surgical procedure performed on the console can be performed in the same way as during normal laparoscopic surgery, and we believe that the procedure was performed safely without any problems. We think that the fact that the 3D vision makes it easier to recognize the context during the dissection around the blood vessel and that the “tactile sense” prevents excessive force from being applied to the organ is also a factor that succeeded in the first surgery.

## Conclusions

We report the first Senhance-assisted ileocecal resection for colorectal cancer in Japan. In the future, we would like to find more ways to use it by accumulating more cases.

## Data Availability

Data sharing is not applicable to this article, since datasets were neither generated nor analyzed for the case series.

## Abbreviations

ASA: American Society of Anesthesiologists
COPD: Chronic obstructive pulmonary disease
CT: Computed tomography
PS: Performance status
